# Metabolites and Immune Response in Tumor Microenvironments

**DOI:** 10.3390/cancers15153898

**Published:** 2023-07-31

**Authors:** Salvatore Cortellino, Valter D. Longo

**Affiliations:** 1 Laboratory of Pre-Clinical and Translational Research, IRCCS-CROB, Referral Cancer Center of Basilicata, 85028 Rionero in Vulture, Italy; salvatore.cortellino@crob.it; 2 IFOM, The AIRC Institute of Molecular Oncology, 20139 Milan, Italy; 3 Longevity Institute, Davis School of Gerontology, University of Southern California, Los Angeles, CA 90089, USA

**Keywords:** immune system, diets, metabolism

## Abstract

**Simple Summary:**

Cancer cells alter their metabolisms to support growth, thus causing micro- and macronutrient changes in the metabolite profile that promote an immunosuppressive tumor microenvironment. Immune cells and stromal cells contribute to an immunosuppressive microenvironment by releasing metabolites, cytokines, and depositing extracellular matrix that prevents lymphocyte activation and recruitment and promotes T-cell exhaustion. In recent years, new dietary interventions such as calorie restriction and periodic fasting have been shown to have beneficial effects in preventing cancer and extending life expectancy in mouse models through modulation of immune system functions. In this review, we discuss how dietary interventions such as periodic fasting can manipulate tumor metabolism, prevent the formation of an immunosuppressive microenvironment, and enhance the efficacy of anti-tumor therapies.

**Abstract:**

The remodeled cancer cell metabolism affects the tumor microenvironment and promotes an immunosuppressive state by changing the levels of macro- and micronutrients and by releasing hormones and cytokines that recruit immunosuppressive immune cells. Novel dietary interventions such as amino acid restriction and periodic fasting mimicking diets can prevent or dampen the formation of an immunosuppressive microenvironment by acting systemically on the release of hormones and growth factors, inhibiting the release of proinflammatory cytokines, and remodeling the tumor vasculature and extracellular matrix. Here, we discuss the latest research on the effects of these therapeutic interventions on immunometabolism and tumor immune response and future scenarios pertaining to how dietary interventions could contribute to cancer therapy.

## 1. Introduction

The effectiveness of anti-tumor therapies is determined by cancer cell-intrinsic factors such as the genomic landscape and cell signaling, as well as by the connective tissue, vessels, fibroblasts, and immune cell infiltration that compose the tumor microenvironment (TME) and contribute to the generation of immunosuppressive environment and immune evasion [1,2,3].

Tumor cells determine TME cell composition through the release of chemokines and cytokines that attract certain cell populations and manipulate TME cell function and differentiation through direct cell–cell interactions or through the release of growth factors, cytokine, and metabolic byproducts.

In recent decades, cancer metabolism has emerged to play an essential role in shaping the TME composition and the properties and functions of its constituents [4]. Dietary interventions such as calorie restriction (CR), fasting, and fasting mimicking diets (FMD) have received much attention as several preclinical and clinical studies have shown their efficacy in modulating tumor metabolism, remodeling the tumor microenvironment, and even enhancing the antitumor immune response [5,6,7,8,9,10,11].

This review focuses on the current knowledge regarding cancer metabolism and how it will affect the TME. We will then examine how dietary interventions can affect cancer metabolism, reverse immunosuppressive TME, and improve immune response.

## 2. Immunosuppressive TME

The TME contains mesenchymal stroma-/stem-like cells (MSCs), cancer-associated fibroblasts (CAFs), cancer-associated adipocytes (CAAs), cancer-associated endothelial cells (CAECs), immune cell populations, and extracellular matrix (ECM), which exert immunosuppressive activity and support tumor cell growth, survival, and spread [12,13,14] through the secretion of signaling molecules and extracellular matrix (ECM) components [15].

MSCs are a heterogeneous population of multipotent stromal cells capable of self-renewal and differentiation into various cell types which support the generation of immune regulatory cells with tolerogenic properties through the production of immunomodulatory factors [16]. Several chemokines secreted by MSC shape the immune cell composition in the TME by influencing leucocytes recruitment, macrophages polarization [17], collagen deposition [18], cancer cell invasion [19], and drug resistance development [20]. Chemokines drive leucocytes trafficking in TME and promote the selective recruitment of specific leucocytes based on their chemokine receptor expression. Thus, chemokines may have both an antitumor role by promoting the recruitment of NK cells, CD4^+^ Th1 cells, and CD8^+^ cytotoxic T lymphocytes (CTLs) into the tumor [21,22,23,24], and a protumor role by promoting the recruitment of immunosuppressive cells such as Tregs [25,26], tumor-associated macrophages (TAMs), and MDSCs [27,28,29]. GRO-y (growth-regulated oncogene) chemokine released from MSC drives monocyte-derived dendritic cells to differentiate into tolerogenic myeloid derived suppressor cells (MDSC) [30]. The secretion of nitric oxide (NO), PGE2, IL-6, IL-10, and kynurenine, generated from tryptophan by indoleamine 2,3-dioxygenase (IDO), induce a switch from the Th1 anti-tumor effector response to Th2 suppressive mode [16], expand immunosuppressive M2-polarized macrophages, tumor-associated macrophages (TAMs), and also Tregs populations [31,32]. Furthermore, the natural killer (NK) activity is impaired by MSC through downregulation of NKG2D-activating receptors expression [33]. MSCs expressing PD-1 by interacting with PD-L1/PDL2 ligand on tumor infiltrating lymphocytes (TILs) induce T cell anergy and exhaustion, thus allowing tumor progression [34].

CAFs are dysregulated fibroblasts which arise from differentiation of tumor stromal cells and are involved in extracellular matrix (ECM) remodeling and immune-surveillance evasion [35,36]. As the most abundant cell population of non-cancerous components in the TME of solid tumors [37], CAFs effectively contribute to the generation of immunosuppressive TME and tumor progression [38]. CAFs support tumor-promoting inflammation [39] by recruiting immune cells to the TME through the release of cytokines and chemokines. Secretion of chitinase-like protein 3 (Chi3L1) by CAFs increases macrophage infiltration, promotes macrophage switch to the M2 immunosuppressive phenotype, and limits the infiltration of cytotoxic CD8^+^ T cells into the breast TME [40]. The expression of fibroblast activation protein (FAP) on the surface of CAF promotes the recruitment of type M2 macrophages, MDSCs, and Tregs into the liver tumor bed and the switch from Th1 to Th2 immunity [41].

CAFs impair innate and adaptive immune responses by downregulating the expression of MHC class II (MHCII) molecules in antigen-presenting cells (APCs) [42], promoting the differentiation of immature dendritic cells (DCs) into regulatory DCs (DCregs) in hepatic carcinoma [43], and stimulating the expression of immunosuppressive proteins such as T cell immunoglobulin and mucin domain-containing protein 3 (TIM-3), CTLA-4, and LAG-3 in active T lymphocytes in pancreatic carcinoma [44].

CAFs contribute to liver tumor cell growth and migration through the secretion of various metabolites in the TME. Glutamine release in TME supports nucleotide synthesis and oxidative phosphorylation (OXPHOS) activity [41]. CAF-secreting alanine is converted to pyruvate, an essential source to fuel OXPHOS when glucose is in short supply in pancreatic tumor cells [45]. The secretion of aspartate and glutamate promotes the synthesis of nucleotides and glutathione, respectively, which is required to reduce oxidative stress in skin cancer cells [46]. The arginine released in the TME is instead utilized for the synthesis of nitric oxide (NO), which is essential to alleviate oxidative stress and promote glycolysis in ovarian and endometrial cancer cells [47]. Finally, CAFs promotepancreatic tumor growth both through the production of lysophosphatidylcholine, essential for the synthesis of cell membranes and of lysophosphatidic acid, a biomolecule with growth factor-like activity [48]; and the deposition of collagen, which is degraded and used by pancreatic cancer cells as a source for the production of proline, which is needed to support the tricarboxylic acid (TCA) cycle [49].

CADs are aberrant adipocytes that promote breast and prostate tumor cell proliferation, angiogenesis, dissemination, invasion and metastasis, and the generation of an immunosuppressive microenvironment through the release of adipokines such as leptin, adiponectin, interleukin (IL)-6, chemokine ligand 2 (CCL2), and chemokine ligand 5 (CCL5) [50,51,52]. Furthermore, tumor cells nourish themselves and support their growth by absorbing lactic acid, fatty acids, and glutamine secreted by CADs in TME [53]. Adipokines exert an immunomodulatory effect and remodel breast TME by favoring the infiltration of immunosuppressive cells such as monocytes, macrophages, and neutrophils and by inactivating the cytotoxic CD8 T lymphocyte [54,55,56].

**Figure 1:**
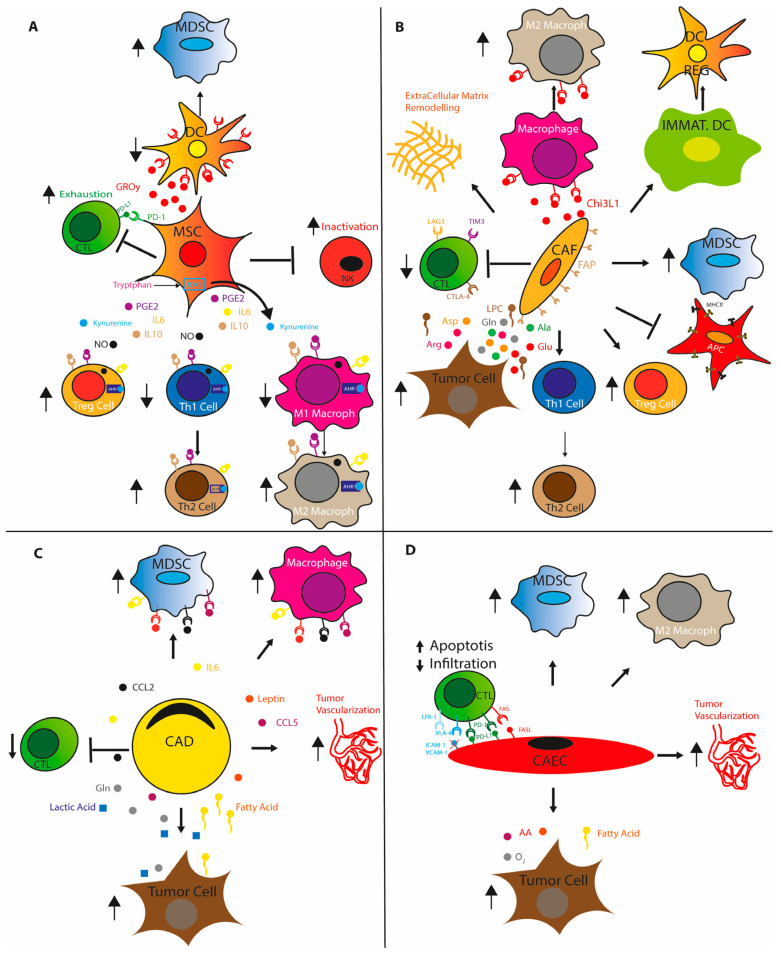
Stromal cells contribute to generate an immunosuppressive tumor microenvironment. (**A**) Mesenchymal stem cells (MSC). (**B**) Cancer associated fibroblasts (CAFs). (**C**) Cancer associated adipocytes (CADs) and (**D**) Cancer associated endothelial cells (CAECs) promote immune suppressive cells recruitment, proliferation and differentiation by releasing chemokines, cytokines and metabolites in TME. Stromal cells induce T cells exhaustion by expressing immune checkpoint and prevents T cell recruitment by reshaping tumor vasculature. IL6, IL10: interleukin 6, 10; PGE2: prostaglandin E2; ID01: Indoleamine-pyrrole2,3-dioxygenase; AHR: Aryl Hydrocarbon Receptor; GRO-y: Growth-regulated oncogene-Y chemokine; Chi3L1: chitinase-like protein 3; Gin: glutamine; Glu: glutamate; Arg: arginine; Ala: alanine; Asp: aspartate; LPC: lysophosphatidylcholine; MDSCs: myeloid-derived suppressor cells; CTLs: cytotoxic T lymphocytes; DCs: dendritic cells; DCregs: regulatory DCs.

CAECs support breast and melanoma tumor growth both by promoting tumor vasculature essential for nutrient and oxygen delivery and by favoring the recruitment of immunosuppressive rather than effector immune cells through the downregulation of adhesion molecules such as the ligands ICAM1 (intercellular adhesion molecule 1) and VCAM1 (vascular cell adhesion protein 1), which promote T cell extravasation into the TME by binding to LFA1 (antigen 1 associated with lymphocyte function) and VLA4 (very late antigen 4) on T cells [57]. Furthermore, CAEC expressing PD-L1 binds PD-1 and inhibits tumor infiltrating lymphocytes (TIL) activation, while the interaction of FASL, expressed on CAEC, with FAS, present on T cell, induces lymphocyte apoptosis [58,59]. Tumor vessels are dysfunctional, abnormal, and characterized by irregular blood flow, erratic branching, and high permeability/leakiness, which lead to an accumulation of metabolic waste in the TME that affects immune cell function [60] (Figure 1).

An immunosuppressive role in TME is exerted by both innate immune system cells (MDSC, TAM, γδ T cells) and by the adaptive system (Bregs, Tregs, Th2, γδ T cells).

MDSCs are immunosuppressive cells that originate from neutrophils or monocytes attracted to TME via the chemokine receptors CXCR2 and CXCR4 [30,34]. In fact, two populations of MDSCs are distinguished: polymorphonuclear (PMN) of neutrophilic origin and monocytic, which derive from monocytes (M). MDSC inhibit immune responses mediated by T cells, B cells, and natural killer (NK) cells through upregulation of signal transducer and activator of transcription 3 (STAT3) expression, induction of ER stress, expression of arginase1, and expression of S100A8/A9 in gastric cancer [61]. However, PMN-MDSCs exert immunosuppressive activity through the production of reactive oxygen species (ROS), peroxynitrite, arginase 1, and prostaglandin E2 (PGE2), whereas M-MDSCs preferentially utilize nitric oxide (NO), immunosuppressive cytokines such as IL-10 and TGFβ, and the expression of immune regulatory molecules such as PDL1 [62].

TAMs originate from bone marrow-derived monocytic precursors recruited in TME by migratory stimulating factors, such as VEGF, CCL2, CCL5, CSF-1, EMAP-II, endothelin-2, SEMA3A, oncostatin M, and eotaxin, released by breast, pancreas, lung, and glioma tumor cells [27,63,64,65,66,67]. According to TME stimuli, TAMs differentiate into M1 or M2 polarized macrophages [68]. IFN-γ-, lipopolysaccharide-, IL-1β-, TNF-, and/or GM-CSF-activated M1-like macrophages recognize and kill tumor cells via phagocytosis and the release of proinflammatory factors that stimulate the immune system. M2-like macrophages, activated by IL-4, IL-10, IL-13, and/or M-CSF, released by Th2, support breast, lung, colorectal, thymomas, and melanoma tumor growth by secreting growth factors (FGF2, PDGF, and VEGF) [69,70], immunosuppressive factors (iNOS, oxygen radicals or nitrogen species) [71,72,73], pro-angiogenic molecules (VEGF, FGF2, CXCL8, and IL-8), and proteases (MMP2, MMP7, and MMP9) [74,75,76].

However, an important role in tumorigenesis and tumor progression could also be played by tissue-specific macrophages responsible for maintaining tissue homeostasis. It has emerged from recent studies that tissue-resident macrophages differ mainly in their metabolism. In particular, OXPHOS-dependent macrophages are involved in the maintenance and handling of extracellular lipids and cholesterol. These OXPHOS-dependent macrophages acquire a proinflammatory phenotype in obese people and contribute to the generation of an inflammatory state which promotes immune evasion and tumor development [77].

Regulatory B cells arise from the differentiation of B cells, attracted to the tumor upon the release of chemoattractants, and suppress inflammatory responses through the secretion of immunomodulatory cytokines and cell-to-cell contact interactions. Secreted factors such as leukotriene B4 (breast cancer), glioma cell-derived placental growth factor (glioma), TNF-α (tumor cells), and IL-21 (T cells), as well as cell contact-dependent mechanisms (CD40-CD40L and PD-1-PD-L1 axis,), promote cell differentiation Bregs in the TME [78,79,80,81,82,83].

Inflammatory cues such as TLR ligands (CD40L, TLR9signals) and pro-inflammatory cytokines (IL-21, IL-1b, and IL-6; B cell-activating factor and A proliferation-inducing ligand) induce immune suppressor function of Bregs [84].

Bregs exert inhibitory activity mainly through the release of IL10, TGF-b, IL-35, and granzyme B (GzmB), but also mediate immunosuppressive function through the expression of PD-L1, FasL, and TIM-1. Tumors stimulate Bregs to secrete IL10 via CD40L signals or tumor-derived exosome [85,86,87,88]. IL10^+^ Bregs promote immune evasion by suppressing IFN-g release by effector CD8^+^ and CD4^+^ T cell and activated NK cells, by reducing GzmB synthesis in CD8^+^ T cells, by inhibiting the secretion of pro-inflammatory cytokine by CD4^+^ T cells, and by hampering CD20-mediated lymphoma clearance by preventing macrophage activation [85,87,89].

Bregs promote breast tumor progression and metastasis by producing and releasing TGF-b in TME. TGF-b convert naïve CD4^+^ T cells into Tregs and inhibit CD4^+^ and CD8^+^ T cell proliferation by inducing reactive oxygen species and NO production in MDSC [90,91].

Upon BCR and IL21 stimulation, Bregs produce GzmB which suppress CD4^+^T cell proliferation and Th1 and Th17 response by degrading z-chain [81]. Furthermore, Bregs support pancreas and gastric cancer by secreting IL35, which is a potent anti-inflammatory cytokine produced mainly by Tregs cells [92,93].

In pancreatic breast and cervical cancer, the engagement of PD1 with PD-L1, expressed on Bregs cells surface, induces CD4^+^ and CD8^+^ T cell suppression and exhaustion, IL-10-secreting type-1 regulatory CD4^+^T cells (Tr1) expansion, and reduced Th1/Th17 responses [94,95,96].

Bregs induce activated T cells anergy or apoptosis by expressing FasL on the cell surface. The interaction of FasL with Fas expressed by activated T cells leads to activation-induced cell death, a common apoptotic pathway [97,98]. Finally, Bregs promote tumor immune escape by expressing TIM-1 receptor, which is involved in regulating suppressive cytokines and ligands expression signaling pathways. The binging of TIM-1 with its ligand (TIM-4) expressed by myeloid cells leads B cells to produce IL10, TGF-b, and promote Treg and Tr1 generation, CD4^+^ and CD8^+^ T cell suppression, and inhibition of Th1 and Th17 differentiation [83,99,100].

Recently, it has been shown that Bregs mediate immunosuppressive function though the release of adenosine, IDO, progesterone-induced blocking factor 1, and heat shock protein-70 [101,102,103,104,105].

Treg and Th2 cells are derived from CD4 differentiation induced by tumor microenvironmental signals, such as tumor antigens, cytokines (such as TGF-β), and other soluble molecules [106], and have strong immunosuppressive, antitumor immunity inhibitory function [107,108,109].

Th2 cells contribute to fibrosarcoma and lung tumor progression by secreting IL-4, IL-5, IL-10, IL-13, and IL-17 [69,70,110,111] and promoting the recruitment of M2 macrophages and eosinophils via the expression of IL-5 and IL-13 [112]. Furthermore, Th2 cells promote tumor cell migration and invasion by releasing IL17, a pro-angiogenic factor, which induces vascular leakage [113] and increases MDSC infiltration.

Tregs promote tumor progression by impairing the functions of effector T cells (Teff), NK cells. and DCs by (1) secreting immunosuppressive cytokines, such as IL-10, TGF-β, and IL-35 [114]; (2) killing effector T cell through the release of granzymes and perforin; (3) regulating memory T cell quiescence through the expression of inhibitory receptor CTLA-4 [115]; (4) expressing CD39 and CD73 nuclease which increase the production of adenosine in TME, a molecule with antiproliferative effects [116]; and (5) hampering DC activation through the expression of activation gene 3 (LAG3), CTLA-4, and IDO [117].

γδ T cells represent a small sub-population poised at the border between the innate and adaptive immune system that can mediate an anti- or pro-tumor response based on the differentiation status. γδ Tcells producing effector cytokines IFNγ kill tumor cells via perforin and granzyme, whereas γδ T cells expressing IL17 support tumor growth and metastasis by recruiting other immunosuppressive immune cells, such as MDSC [118].

ECM is composed primarily of collagens, hyaluronan, fibronectin, elastin, and laminins, and constitutes 60% of tumor mass [119]. The tumor extracellular matrix is denser and stiffer than that of normal tissue [120], a characteristic that supports tumor growth by preventing drug diffusion and hindering the infiltration of T lymphocytes and NK cells into the tumor [121]. Dense ECM affects tumor immunosurveillance by collapsing lymph vessels and hindering migration of antigen-triggered DCs to draining lymph nodes and consequently antigen presentation and T-cell activation [122].

Tumor cells select and recruit TME cells through the release of hormones and cytokines and modulate their immunosuppressive activity through surface receptor–ligand interaction or secretion of hormones and metabolites.

Myeloid derived suppressor cells (MDSCs) and immunosuppressive regulatory T cells (Tregs) are recruited to the tumor region [123] by TGF-β1 (transforming growth factor beta-1) and granulocyte–macrophage colony-stimulating factor (CSF) secreted by tumor cells. CD47 expression on tumor cell membrane suppresses the antitumor activity of macrophages by inhibiting the phagocytosis through the interaction with SIRPα (signal regulatory protein α) [124]. The production of immunosuppressive metabolite kynurenine due to the upregulation of indoleamine-2,3-dioxygenase (IDO1) in tumor, tumor associated macrophages (TAMs), and monocytes prevents the activation of effector T cells, inhibits NK cell function, supports Treg activation, and promotes the expansion and activation of dendritic cells (DCs) and MDSCs [125].

**Figure 2:**
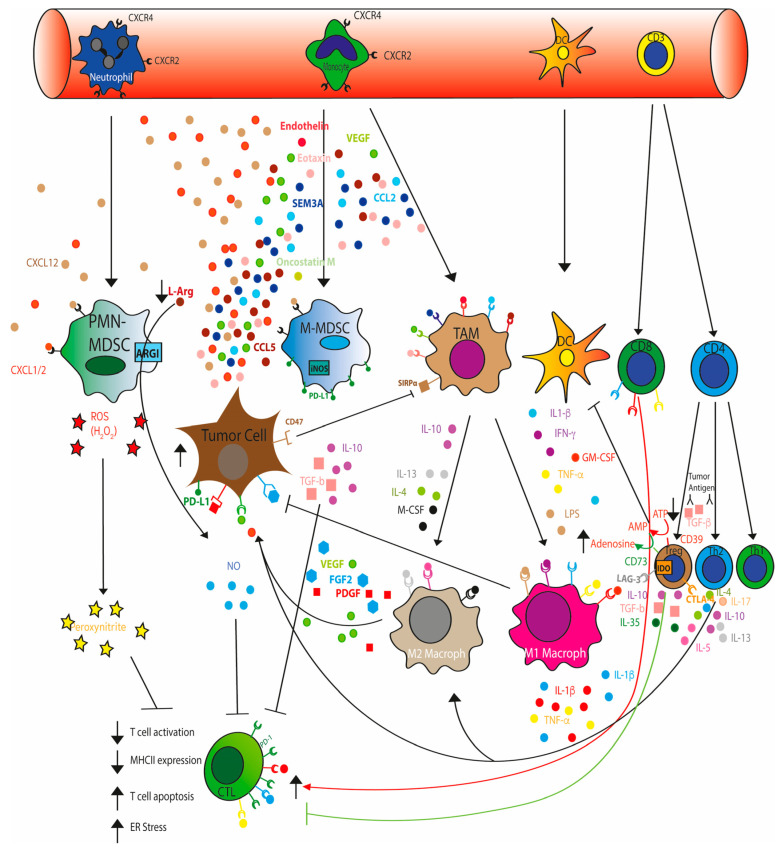
Cancer cells shape the tumor microenvironment through secretion of soluble factors and recruitment of immunosuppressive cells. Neutrophils and monocytes migrate to TME attracted by chemokines such as CXCL12 and CXCL1, released by cancer cells, and differentiate into polymorphonuclear (PMN−) and mononuclear (M−) MDSCs. PMN-MDSCs suppress immune cells by producing ROS, peroxinitrite and prostaglandins and depleting TME of arginine by upregulating arginase I expression. M-MDSCs inhibit T cells, B cells and natural killer (NK) cells by producing nitric oxide (NO), immunosuppressive IL-10 and TGFβ and immune checkpoint molecules such as PDL1. Chemokine and growth factors (VEGF, CCL2, CCL5, CSF-1, EMAP-II, endothelin-2, SEMA3A, oncostatin M, and eotaxin), secreted by cancer cells, promote the migration of monocytes into the TME and their differentiation into tumor-associated macrophages (TAMs). TAMs differentiate into anti-tumor M1 macrophages upon stimulation with IFN-γ, lipopolysaccharide, IL-1β, TNF and/or GM-CSF, and into pro-tumor M2 macrophages upon stimulation with IL-4, IL-10, IL-13 and/or M-CSF. Tumor antigens, cytokines (such as TGF-B) promote CD4 differentiation in immunosuppressive Treg and T helper type 2 cells (Th2). Th2 cells secrete protumor cytokines IL-4, IL-5, IL-10, IL-13, and IL-17, recruits M2 macrophages by releasing IL-5 and IL-13 and promotes MDSCs infiltration by increasing vascular leakage through IL17 secretion. Tregs impair CTL, NK and dendritic cell function by secreting immunosuppressive cytokines such as IL-10, TGF-β and IL-35, expressing the immune checkpoint receptor (LAG3, CTLA4) the enzyme IDO, which converts tryptophan to kynurenine and CD39 and CD73 nucleases that convert ATP to adenosine.

Furthermore, tumors cells, MDSCs, monocytes, and TAMs promote immunosuppressing microenvironment by expressing the immune checkpoint PD-L1, which induces the T cells exhaustion and anergy by binding to PD1 [31,126] (Figure 2).

Recent data indicate that NK cells and PGE2 play a fundamental role in reprogramming TME towards a cancer-promoting versus cancer-inhibitory condition which affects the anti-tumor immune response and the efficacy of immune therapy. The early intratumoral accumulation of natural killer (NK) cells producing interferon gamma (IFN-g), drives a broad polarization of myeloid cells toward an inflammatory profile conducive to effector T-cell infiltration. In contrast, tumor-secreted PGE2 in TME inhibits NK cells by binding to EP2 and EP4, the PGE2 receptors, and thus hinders NK-induced TME switch and promotes immune evasion of melanoma, breast, and colon cancer [76].

## 3. Cancer Metabolism

Cancer cells accumulate mutations in oncogenes and tumor suppressor genes leading to the constitutive or increased activation of signaling pathway which renders the tumor cells insensitive to or partially independent of growth factor stimuli and nutrient availability, leading to proliferation [127]. To support cell proliferation and meet the biosynthetic and bioenergetic requirements for growth, cancer cells require higher amounts of specific nutrients compared to healthy cells [128]. These high-nutrient demands change the metabolic landscape in the TME, and result in areas deprived of certain nutrients, including glucose and amino acids [129]. A nutrient-restricted TME impairs T cell differentiation and function while promoting the expansion and potentiating the suppressive capacity of immunosuppressive immune cells [130].

Most cancers prefer aerobic glycolysis over oxidative phosphorylation (OXPHOS) because even though it is a low-efficiency process for energy generation, it produces large amounts of glycolytic intermediates essential for the biosynthesis of biomass molecules and molecules involved in the cell’s redox state control [131,132].

Furthermore, aerobic glycolysis supports the biosynthesis of proteins, heme, and nucleotides by promoting the synthesis of serine from glucose through the activity of phosphoglycerate dehydrogenase (PHGDH) [133,134]. At the same time, PHDC, by generating 3-phosphohydroxypyruvate (3PHP), stimulates the conversion of glutamate to α-ketoglutarate (a-KG), which supplies the dietary tricarboxylic acid (TCA) cycle and allows pyruvate-derived citrate to be shuttled from the mitochondria to the cytosol where it is converted in oxaloacetate and acetyl-coA by ATP citrate lyase (ACLY) [135]. In the cytosol, acetyl-coA is mainly used for fatty acid synthesis [136,137].

The high metabolic demand of tumor cells reduces citrate concentrations in tumor cells, TME, blood, and urine of patients with multiple tumors [138].

High glucose consumption in the tumor leads to lactate secretion as pyruvate produced by aerobic glycolysis is converted to lactic acid by lactate dehydrogenase in order to sustain glycolysis through NAD^+^ regeneration [139]. Lactate accumulation in TME and lactate dehydrogenase (LDH) expression are associated with poor prognosis and survival in patients with various cancers, including breast cancer, gastrointestinal cancer, urogenital cancer, lung cancer, sarcoma, melanoma, myeloid malignancies, lymphoid malignancies, and other types of cancer [140]. High lactate concentrations cause acidification of TME leading to TIL depletion, infiltration of tolerogenic cells such as MDSCs, and, in turn, immune evasion and tumor progression [140,141].

After glucose, glutamine is the main source of energy for several types of cancers, so much so that some cancers are considered glutamine dependent. Glutaminase (GLS) catalyzes the breakdown of glutamine into ammonia and glutamate. Ammonia is utilized for the synthesis of purines and pyrimidines [142,143], whereas glutamate contributes to the production of ATP by entering the TCA cycle as α-ketoacids, to the synthesis of biomass in particular of lipids and other non-essential amino acids, and to the production of glutathione, an antioxidant essential for redox homeostasis [144,145].

Finally, glutamate contributes to aspartate synthesis, which is crucial for cancer cell proliferation, specifically for those with a severely dysfunctional electron transport chain (ETC), as it provides electrons for oxidative phosphorylation (OXPHOS) via the malate-aspartate shuttle (MAS) [146,147].

The dependence of tumors on glutamine also depends on the concentration of cystine. Cystine is essential for glutathione synthesis and thus redox homeostasis and is transported into the cell via the cystine–glutamate antiporter xCT/SLC7A11, which exchanges an extracellular cystine molecule for an intracellular glutamate molecule. This exchange causes a depletion of glutamate, which is produced through the glutaminolysis of glutamine [148].

Most cancer cells depend on methionine, an essential amino acid involved in glutathione and protein synthesis and DNA methylation. Methylthioadenosine phosphorylase (MTAP) [149] and methylenetetrahydrofolate reductase (MTHFR) defects result in methylthioadenosine accumulation, methyltetrahydrofolate deficiency, and impairment of the methionine salvage pathway, essential for the regeneration of methionine from methylthioadenosine (MTA) and for the production of polyamines critical for cell proliferation [150,151]. Deficiency of the cofactors cobalamin and folate, crucial for the production of tetrahydrofolate and the replacement of cysteine by homocysteine, not convertible in methionine due to defects in cysteine uptake, contribute to making tumor cells dependent on methionine [152,153].

Cancer cells are addicted to external arginine, as they are unable to synthesize it because they do not express arginine synthase 1 (ASS1), which, in combination with argininosuccinate lyase (ASL), synthesizes arginine in normal cells from citrulline and aspartate. In order to meet the high arginine demand, cancer cells overexpress several arginine transporters such as SLC6A14, SLC7A3, and SLC7A9.

Arginine is a non-essential amino acid involved in the synthesis of nitric oxide (NO) and polyamines [154,155], mTOR activation, and epigenetic remodeling. NO plays a pro-tumorigenic role by promoting angiogenesis, metastasis, and inhibiting apoptosis [156]. Arginine-activated mTOR is implicated in tumor cell growth and proliferation but also epigenetic reprogramming [157]; mTOR promotes acetyl-CoA synthesis through the activation of ATP citrate synthase (ACLY) and Acyl-coA synthetase short-chain family member 1–2 (ACSS 1–2) [158]. Increased acetyl-CoA promotes histone acetylation via the activation of histone acetyltransferases (HATs) [159].

Serine and glycine play important roles in tumor growth as they are involved in nucleotide synthesis and DNA methylation. Cancer cells can meet their need for serine by absorbing it from the extracellular matrix or synthesizing it de novo from glucose via the de novo serine synthesis (SSP) pathway [160]. De novo serine produced via the SSP pathway promotes and sustains the activation of glycolysis by binding to pyruvate kinase M2 (PKM2), the last rate-limiting enzyme of glycolysis [161,162]. The conversion of serine to glycine by serine hydroxymethyltransferase (SHMT1-2) generates 5,10-methylene-THF essential for purine and pyrimidine synthesis [163,164]. Furthermore, serine and glycine influence DNA/RNA methylation and de novo ATP synthesis in cancer cells through the one carbon pathway [165].

Leucine, isoleucine, and valine belong to the branched chain amino acids (BCAA) and are essential amino acids obtained only via food intake [166]. BCAA are transported via lat1(SLC7A5), a transporter that imports several essential amino acids (e.g., phenylalanine, leucine, isoleucine, tryptophan, histidine, and tyrosine) in exchange for intracellular histidine, tyrosine, and glutamines [167,168]. Lat1 is highly expressed in cancer cells as its transcription is regulated by oncogenes such as c-Myc [169], HIF2a [170], and NOTCH [171]. BCAAs are catabolized into glutamate and branched-chain α-ketoacids (BCKA), which are converted to TCA intermediates and provide acetyl-CoA and/or succinyl-CoA essential for energy production [172] or acetylation modification [173].

## 4. TME Metabolism

The different cell lines that make up TME have distinct metabolisms that may depend on cellular activation status and the availability of specific nutrients in the microenvironment. Highly proliferative CD4- and CD8-activated T cells, activated B cells, γδIFNγ cells, cancer cells, activated DCs, M1-like TAMs, and intratumoral myeloid cells rely heavily on glucose and glutaminolytic metabolism, preferentially using aerobic glycolysis over TCA-coupled OXPHOS for ATP production [174,175,176].

Glycolysis supports CD8 T cell and B cells’ effector function and interferon-gamma (IFNγ) production via glyceraldehyde 3-phosphate dehydrogenase (GAPDH), LDHA, and mTOR regulation [177,178,179,180,181], whereas glucose promotes CD4 activation and their antitumor activity by sustaining the production of the glycolytic intermediate phosphoenolpyruvate (PEP) which restores Ca2^+^—the nuclear factor of activated T cells (NFAT) signaling [138].

Since different cell lines require glucose to support their function, high glucose consumption can lead to glucose depletion in the TME. Glucose deficiency may induce a metabolic shift towards FA uptake and OXPHOS metabolism in CD8 T cells via PPAR-α signaling pathway activation [182] but may also make them metabolically inactive and exhausted [183]. In addition, glucose deprivation promotes differentiation of effector T cells into Treg cells by inducing Foxp3 expression and thus contributes to the establishment of an immunosuppressive environment [184].

**Figure 3:**
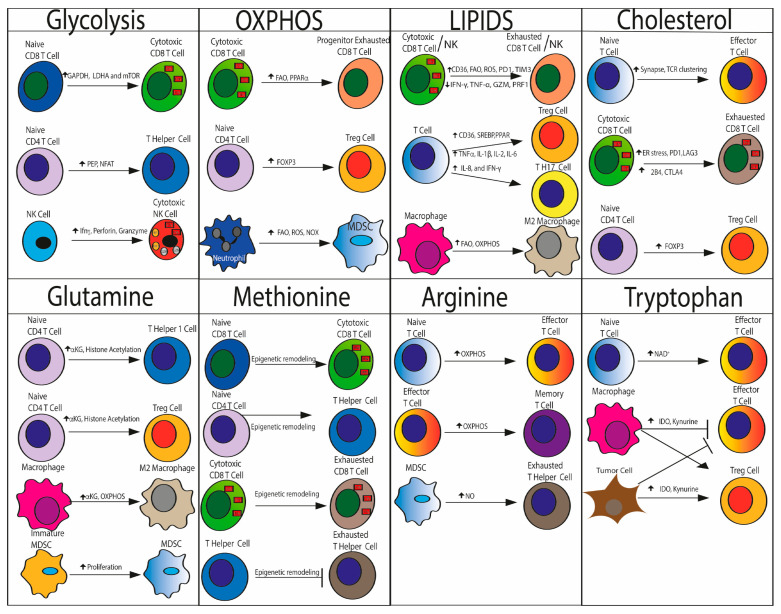
Metabolic changes in TME regulates adaptive and innate immune system function. Glycolysis supports naïve CD8 and CD4 T cells and NK cell activation. Progenitor exhausted T cells, Tregs and MDDSC rely on oxidative phosphorylation (OXPHOS) and fatty acid oxidation (FAO) Increased concentration of FFAs within TME leads to CD8 T cell and NK exhaustion, renders T cell exhausted and anergic, and promotes the differentiation of CD4 T cells into Tregs and Th17 and skews macrophage polarization toward protumoral M2-like TAMs which mainly depend on FAO and OXPHOS for their metabolism and bioenergetic demands. Glutamine metabolism promotes CD4 T cell differentiation towards Th1 and Treg cells, TAM polarization towards the protumor M2 phenotype and MDSC expansion. Methionine metabolism regulate T cell activation and exhaustion by remodeling epigenetic landscape. Arginine enhances T cells survival and supports effector and memory T cells generation by modulates OXPHOS activity, whereas production of nitric oxide from arginine catabolism by MDSC-expressed arginase leads to Thelper cell dysfunction and anergy. Tryptophane is required for T lymphocyte effector functions as promotes NAD^+^ synthesis. Conversion of tryptophan to kynurenine via IDO1, expressed by tumors and macrophages, promotes Treg expansion and renders effector T cells exhausted. Cholesterol improves cytotoxic function by enhancing immunological synapse formation and maturation, and promoting TCR clustering and signaling. Instead, high cholesterol content in TME hampers T cell function as induces ER stress and expression of exhaustion markers, such as PD-1, LAG-3, TIM-3, 2B4 and CTLA-4. Finally cholesterol induces FOXP3 expression and promotes Treg differentiation. LDHA: lactate dehydrogenase A; GAPDH: glyceraldehyde-3-phosphate dehydrogenase; PEP: phosphoenolpyruvate; NFAT-1: nuclear factor of activated T cells; lfnγ: interferon-γ; PRF1: perforin; GZM: granzyme B; α-KG: α-ketoglutarate; NO: nitric oxide; OXPHOS: oxidative phosphorylation; FAO: fatty acid oxidation; ROS: reactive oxygen species; NOX: NADPH oxidase isoforms.

Tumor-infiltrating MDSCs, unlike peripheral MDSCs that rely on glycolysis, exhibit an increased FAO and OXPHOS metabolism [185,186]. Under these TME conditions, neutrophils support the production of ROS by NADPH oxidase (NOX), which potently suppresses innate and adaptive immunity, adopting a metabolism based on mitochondrial oxidative metabolism and fatty acid oxidation (FAO) [187]. The cytotoxic activity of NK cells also depends on glucose metabolism; thus, the lack of glucose impairs the production and release of Ifng and cytotoxic perforins and granzymes [188] (Figure 3).

High glucose consumption by myeloid cells, T cells, B cells, and tumor cells leads to an elevated lactic acid release which affects the function of various immune cells and reduce T cells antitumor efficacy [189,190].

Lactate accumulation reduces effector T cell proliferation and cytotoxicity by reducing IL-2 and IFN-γ production and lytic granule exocytosis through impairment of TCR-triggered phosphorylation of JNK, C-Jun, and p38, and inhibition of STAT5 and ERK activation and mTOR inhibition [141,191].

**Figure 4:**
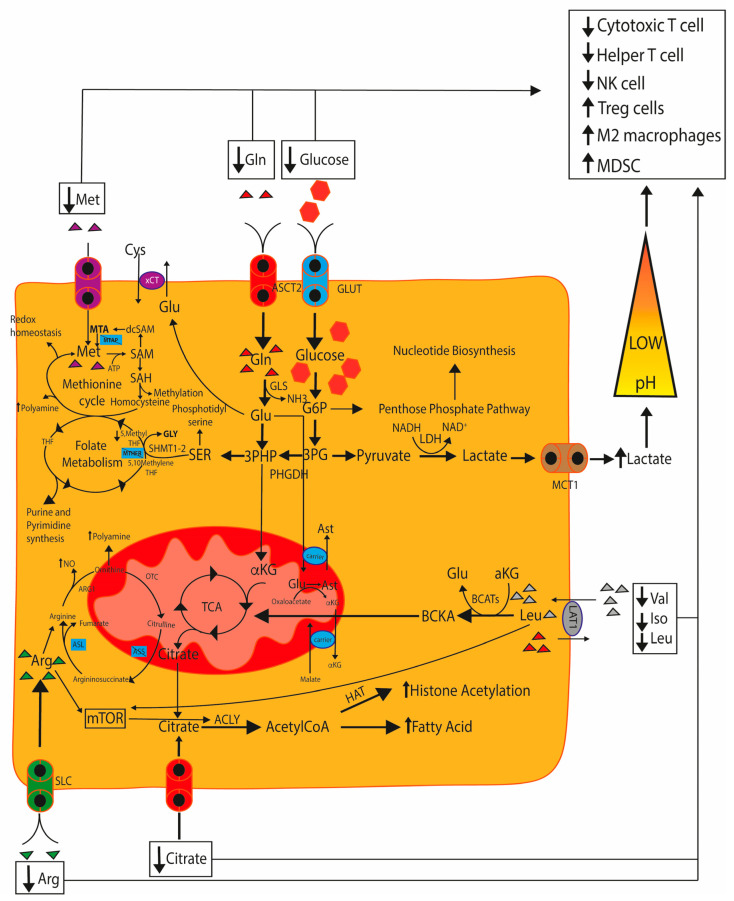
Cancer metabolism promotes immunosuppressive TME. Cancer cells mainly rely on glycolysis as it supports the biosynthesis of nucleotide, via penthose phosphate pathway and folate metabolism cycle, and molecules involved in controlling the redox state of the cell, via methionine cycle. LDH: lactate dehydrogenase; 3PG: 3-phosphoglyceric acid; 3PHP: 3-phospho-hydroxypyruvate; Glu: glutamate; α-KG: α-ketoglutarate; TCA: tricarboxylic acid cycle; ACLY: ATP citrate lyase; OXPHOS: oxidative phosphorylation; MAS: malate-aspartate shuttle; xCT: Cystine/Glutamate Antiporter; MTAP: methylthioadenosine phosphorylase; MTHFR: methylenetetrahydrofolate reductase; ASS1: arginine synthase 1; ASL: argininosuccinate lyase; Arg: arginine; SLC6A14, SLC7A3, SLC7A9: arginine transporters; NO: nitric oxide; ARG1: arginase 1; Lat1: branched amino acids transporter; BCKA: branched-chain α-ketoacids.

Low pH in TME, due to lactic acid release, inhibits monocyte and NK cell activation, dendritic cell differentiation, and promotes Th17 cell differentiation [192], MDSCs accumulation, and macrophages M2 polarization through arginase I [193,194,195,196]. Increased arginase I expression in macrophages due to acid lactic accumulation in TME leads to depletion of arginine, an essential amino acid for effector T cells activation and proliferation [197] (Figure 4).

Finally, lactate promotes angiogenesis and immune evasion through the activation of G protein coupled receptor (GPR81) on immune cells and endothelial cells [198].

The reduction of citrate in TME due to high consumption by tumor cells influences the function and regulation of immune cells. The conversion of citrate to acetyl-CoA by ATP citrate lyase (ACLY) is essential for histone acetylation and thus for epigenetic reprogramming. Thus, citrate depletion affects epigenetic reprogramming, thereby impairing memory CD8^+^ T cell recall responses and CD4^+^ helper T cell (Th1 cell) proliferation and activation, while promoting Th17 viability at the expense of the Tregs [199,200] (Figure 4).

Since glucose is scarce in the tumor microenvironment, glutamine can become the primary energy source. Glutamine metabolism supports T and B cell activation and promotes CD4 T cell differentiation towards Th1 and Treg cells through the production of α-ketoglutarate essential for histone acetylation and epigenetic remodeling [201,202]. In addition, glutamine-derived α-ketoglutarate promotes MDSC expansion and TAM polarization towards the protumor M2 phenotype [203].

However, inhibition of glutamine metabolism promotes the immune response of effector T cells by switching metabolism towards glycolysis, slowing tumor growth by reducing both glycolysis and oxidative phosphorylation and increasing anti-tumor M1 polarized macrophages [204] (Figure 3)

Methionine metabolism is also critical for T cell function and differentiation as it regulates the epigenetic landscape through histone and DNA methylation [205]. Lack of methionine and S-adenosylmethionine due to the high consumption by cancer cells leads to CD4 and CD8 T cells dysfunction and exhaustion, inhibition of Th17 proliferation, and cytokine production [205,206].

Arginine is an amino acid valuable for the CD8^+^ T cells survival, antitumor response, and for the formation of memory T cells [207]. However, the high uptake of arginine by tumor cells, the catabolism of arginine by MDSC-expressed arginase [208,209], and the conversion of arginine to nitric oxide by macrophage-expressed nitric oxide synthetase [195,210] result in arginine depletion in the TME leading to T cell dysfunction and anergy.

Tryptophan and its metabolism also play an important role in generating a pro- or anti-tumorigenic microenvironment. Expression of IDO1 by tumor, Bregs, and TAM cells promotes the conversion of tryptophan to kynurenine, thus resulting in tryptophan deficiency in TME. Tryptophan is essential for T cell function, while kynurenine inhibits CD8 effector T cells by blocking mTOR signaling pathway and promoting Treg activation, MDSC expansion, and TAM M2 polarization [125,211,212]. Thus, tryptophan depletion and elevated kynurenine repress T cell responses and promote immune evasion [213].

At low concentrations, lipids have a beneficial effect on T cell proliferation and activation, while at high concentrations in TME, they promote immunosuppression and T lymphocyte apoptosis [214]. In a hypoglycemic environment, CD8 T cells adapt their metabolism by increasing the expression of genes such as PPAR-a and CD36 involved in fatty acid beta oxidation and uptake [203].

However, the accumulation of fatty acids increases oxidative phosphorylation metabolism in CD8 T cells and reduces the expression of very long chain acyl-CoA dehydrogenase (ACADVL), the first enzyme of fatty acid beta oxidation, which determines the accumulation of very long fatty acid, which leads to mitochondrial dysfunction, high reactive oxygen species (ROS) production, metabolic exhaustion, apoptosis, and ferroptosis [215,216,217]. FA uptake by CD36 induces PD-1 and TIM-3 expression, reduces IFN-γ and TNF-α production, and leads to CD8 terminal exhaustion [218].

In addition, fatty acids stimulate T lymphocytes to secrete pro-inflammatory cytokines, such as TNFα, IL-1β, IL-2, IL-6, IL-8, and IFN-γ, and promotes Treg differentiation, the generation of immunosuppressive and protumor TME [219,220]. Treg and Th17 cells, major immunosuppressive population, rely on FAO and OXPHOS metabolism to support their survival and immunosuppressive function [221]. To meet their high need for fatty acids, Tregs increase the expression of CD36 and SREBP, the major regulatory gene of de novo cholesterol and fatty acid synthesis, and induce PPAR-dependent lipid metabolism [13,222]. FA enrichment in TME leads TAM to shift their metabolism toward FAO and OXPHOS and promotes TAM polarization to immunosuppressive M2 phenotype [223]. FA affect NK cytotoxic activity, as high FA levels inhibit the protein complex mTORC1, essential for IFN-γ and granzyme B production [223].

In the breast, melanoma, colorectal tumor microenvironment dendritic cells, accumulate fatty acids because they overexpress the scavenger receptors (SR) involved in the intracellular transport of lipids. High levels of fatty acids impair the DC capacity to process and present antigen and their ability to stimulate and activate T cells [224].

Cholesterol is essential for T lymphocyte function as it enhances immunological synapse formation and maturation, promotes TCR clustering and signaling, and thus improves cytotoxic function [184,225]. However, high cholesterol content in TME impairs T cell function as it impairs lipid metabolism and induces ER stress leading to increased expression of exhaustion markers, such as PD-1, LAG-3, TIM-3, 2B4 and CTLA-4, on CD8^+^ T cells [226]. The accumulation of cholesterol esters, transported by CD36, leads to ferroptosis of CD8 T cells. Cholesterol oxidation by CYP27A1 generates oxysterol, which promotes tumor cell proliferation and the recruitment of immunosuppressive cells such as neutrophils and IL17-expressing γδ cells [226].

Finally, cholesterol induces FOXP3 expression and promotes Treg differentiation, thus supporting the generation of immunosuppressive TME [227] (Figure 3).

## 5. Fasting, Caloric Restriction, Metabolite Restriction, and Fasting Mimicking Diet (FMD)

Calorie restriction is a diet that involves a daily reduction (10–30%) of caloric intake for an extended period of time. Fasting, on the other hand, consists in complete abstinence from food, but not from water, for a limited period (from 16 h up to a few days) and is not easily applicable in the clinic as it is very restrictive and poorly tolerated by patients. For this reason, a new dietary intervention called “fasting-mimicking diet” has been developed, a 5-day food program which consists of a 50% reduction in calorie intake on the first day and a 90% reduction in the following 4 days. Unlike calorie restriction and fasting, FMD is easily tolerated by patients as it alternates a short period of severe calorie restriction with a normal calorie intake diet. The amino acids or metabolites restricted diet provides a normal caloric intake but lacks specific amino acids or metabolites essential for the regulation of signaling pathways, often altered in tumor cells. However, even these diets could have side effects such as causing serious weight loss in patients as is found in diets lacking essential amino acids.

## 6. Effects of Fasting and Metabolite Restriction on Cancer Cells

Food restriction induces systemic and local metabolic changes through the modulation of hormone and nutrient sensing pathways. The reduction of circulating glucose upon dietary restrictions leads initially to glycogenolysis in order to support gluconeogenesis; then, when glycogen storage is depleted, it leads to lipolysis and the release of glycerol and fatty acids essential for the production of ketone bodies, the main energy sources in the absence of external nutrients [8,228]. The organism responds to the macronutrients and particularly amino acid limitation by inhibiting the anabolic pathways through the downregulation of growth hormone (GH) and insulin growth factor (IGF1). The low level of IGF 1 and of amino acid and increased AMP/ATP ratio lead to the inhibition of PI3K and mTOR pathway and to the activation of autophagy, a process that provides energy and substrates by removing the damaged and redundant self-components [229,230].

In normal cells, the effects of CR on the reduction of growth factors is also associated with the upregulation of anti-stress response (NRF2 and antioxidant genes), which can include DNA repair and cause reduced inflammation trough the release of corticosteroids, ghrelin, and adiponectin and the inhibition of pro-inflammatory cytokine secretion [231,232].

The inhibition of the nutrient-sensing pathway (IGF1-PI3K-mTOR), the activation of the antistress response pathway, and the upregulation of genes involved in DNA repair induced by caloric restriction/fasting prompt normal but not cancer cells to slow down their proliferation and enter a quiescent state, an effect called “differential stress resistance” which protects normal but not malignant cells from the cytotoxicity of anti-tumor drugs. However, tumor cells that possess mutations in oncogenes (IGF-1R, Ras, AKT, and mTOR pathways) and tumor suppressor genes (p53, p16, and Rb) are not only insensitive to lack of nutrients and low levels of anabolic hormones and do not become protected during fasting condition, their uncontrolled proliferation and increased requirement for many metabolites can sensitizes them to anticancer therapy. This mechanism induced by fasting in tumor cells is termed differential stress sensitization, since only cancer cells (breast, glioma, neuroblastoma, melanoma) and not normal cells are sensitized to a wide variety of therapies ranging from chemotherapy to radiotherapy to hormone therapy (DSS) [233,234,235,236].

A key effect of fasting is the reduction of carbohydrates, which are the main energy source for many types of cancers. The scarcity of carbohydrates has a particular impact on the metabolism of tumor cells, which, prior to the restriction, adopt an increased glycolytic mode, called the Warburg effect, and which, in response to glucose restriction, attempt to shift from glycolysis to oxidative phosphorylation to generate more energy but also as the fatty acids and ketone bodies generated by lipolysis become a fuel source. However, glycolysis-dependent tumor cells (ovarian, breast, thyroid cancer) are unable to effectively adapt their metabolism to OXPHOS, so this change induces a slowdown in proliferation and an increase in oxidative stress and apoptosis [237,238,239].

The energy deficit induced by caloric restriction/fasting leads to an increase in NAD^+^, which activates histone deacetylase sirtuins. Sirtuins reshape the epigenetic landscape, causing an inhibition of glycolysis and thus reinforcing the inhibitory effect of calorie restriction on tumor glucose metabolism [240,241,242].

Cycles of fasting or fasting-mimicking diets (FMD), developed to simulate the effects of fasting and lasting 2–5 days, also sensitizes glycolysis-dependent cancer cells (TNBC) to glucose analogues (2DG) and affects cancer stem cell self-renewal [243]. Furthermore, the reduction of glucose and circulating IGF enhance the efficacy of cycline kinase inhibitors/hormone therapy combination and PI3 kinase inhibitors against ER-positive and triple-negative tumors, respectively, preventing drug resistance in ER^+^ breast cancers [244] and completely eradicating triple-negative breast tumors [243].

Fasting cycles could affect tumor growth by modulating the levels of essential (leucine, lysine, and methionine) and non-essential (serine, glycine, glutamine, and asparagine) amino acids [245].

For example, serine deprivation activates the serine synthesis pathway and inhibits glycolysis by promoting OXPHOS [165,246]. This metabolic switch increases ROS production, thus sensitizing colorectal tumors, lacking p53, to agents that increase ROS [247]. Fasting/FMD also alters the iron metabolism in KRAS-mutated tumor (pancreas, lung, colorectal) cells by reducing the levels of heme oxygenase and ferritin, thus increasing sensitization to high doses of vitamin C, which acts as a pro-oxidant agent [248].

Methionine restriction also inhibits tumor growth and increases the efficacy of chemotherapies [249,250] and reverses the drug resistance of RAS-driven colorectal cancer as it affects nucleotide metabolism and glutathione synthesis and reactivates the expression of silent tumor suppressor genes by reshaping the epigenetic and DNA methylation landscape [251].

Several cancer cells are glutamine-addicted because glutamine plays an essential role in cell metabolism as it supplements tricarboxylic acid (TCA) cycle and participates in the biosynthesis of nucleotides, glutathione (GSH), fatty acid, and other nonessential amino acids (alanine, proline, aspartate, asparagine, and arginine) [252]. In addition, glutamine support the production of α-KG, which is a cofactor for Jumonji C domain-containing histone demethylases (JmjC), and ten-eleven translocation (TET) family DNA demethylases [253,254]. Therefore, glutamine deprivation affects cancer growth by impairing the energy and nucleotide metabolism and redox homeostasis [255]. However, glutamine depletion leads to histone and DNA hypermethylation through the inhibition of JmjC and TET. These epigenetic changes promote cancer cells dedifferentiation and drug resistance [256].

Fasting mobilizes lipids from adipose tissue as beta-oxidation of fatty acids becomes the main energy source in glucose deficiency. Therefore, the high consumption of lipids at the cellular level allows for a reduction in plasma lipid levels [8]. This metabolic change affects the metabolism of cancer cells and their ability to adapt. In fact, tumor cells dependent on glycolysis can be more affected by this metabolic change as they are not able to effectively process the fatty acids that accumulate in the lipid droplets [257]. Furthermore, CR reduces the activity of the enzyme stearoyl-CoA desaturase (SCD), essential for maintaining the fluidity of the cytoplasmic membrane through the production of monounsaturated fatty acids (MUFA) [238].

On the contrary, the growth of tumor lines, whose metabolism relies on OXPHOS, is less affected by the effects of caloric restriction and fasting, as they support their growth through the beta oxidation of fatty acids, which are reduced in the tumor microenvironment [257].

Calorie restriction and fasting may exert an antitumor action through downregulation of the satiety hormone leptin and upregulation of adiponectin. Fasting blocks the progression of acute lymphocytic leukemia (ALL) through upregulation of the leptin receptor (LEPR). LEPR expression slows cell proliferation and promotes ALL differentiation by upregulating XBP1, involved in the unfolded protein response (UPR) pathway, and PRDM1, the tumor suppressor gene involved in T and B cell differentiation [258]. Calorie restriction has been shown to prevent the risk of radiation-induced myeloid leukemia in mice if performed both before and after irradiation [259]; however, it has no effect on multiple myeloma progression, although it is able to remodel the microenvironment of the bone marrow [260]. Therefore, the antitumor effects of CR and FMD are cancer specific and dependent on the genotypic and phenotypic characteristics of the tumors.

## 7. Effects of Fasting and Caloric Restriction on TME

Although the reduction of glucose and essential and non-essential amino acids has a detrimental effect on tumor growth, such restrictions also negatively impact the function of the immune system. Glucose reduction suppresses proliferation and activation of tumor infiltrating effector immune cells while promoting the formation of long term memory CD8^+^ T cells [261,262]. At the same time, glycine and serine restriction inhibit CD8 T cell activation [263], while low methionine levels impair CD4 T cell activation by reducing histone methylation at the levels of genes involved in T cell proliferation and activation [264].

Although calorie restriction/fasting reduces glucose and amino acids levels, such dietary interventions have been shown to have beneficial effects on the antitumor response by increasing the percentage of CD8^+^ cytotoxic T cells, memory T cells, and stem cell-like memory T cells and repressing Treg through the reduction of IGF1 levels, epigenetic reprogramming, and the production of ketone bodies [257,265,266,267,268,269,270,271].

More recently, fasting/FMD cycles were shown to promote the immune cell-dependent attack of different types of cancer cells (melanoma, breast, gastric cancers) [256,257,269,272,273].

This was attributed to the ability of fasting/FMD to reduce the Treg population and boost CD8 infiltration by downregulating the expression of heme oxygenase-1 (HO-1) and IGF-1 and by inducing autophagy in melanoma and breast cancer [271,272]. FMD potentiates the cytotoxic effect of chemotherapy and increases the immune infiltrate in breast and melanoma cancer by reducing tumor HO-1 expression, a potent immunomodulator involved in suppressing CD8^+^ T-cells infiltration and cytotoxic activity and promoting Treg accumulation [272]. Fasting sensitizes low immunogenic non-small-cell lung cancer lung tumors to anti-PD1 immunotherapy and even leads to complete tumor remission in preclinical studies by reversing or neutralizing the immune evasion mechanism through IGF-I signaling pathway downregulation [271].

Since IGF-1 cooperates with the receptor for advanced glycation end products (RAGE) in inducing metabolic-dependent inflammatory responses, and since RAGE activation amplifies IGF-1 signaling, CR and FMD could improve the antitumor immune response by modulating the RAGE signaling pathway. RAGE activation by advanced glycation end-products and 25 other ligands induces transcription of inflammatory cytokines and chemokines that contribute chronic inflammation in the tumor microenvironment promoting MDSC recruitment, TAM polarization from the proinflammatory M1 to the anti-inflammatory M2 phenotype, DC dysfunction, and T cell exhaustion. Furthermore, RAGE triggers the activation of the NLRP3 inflammasomes pathway, which favors immunosuppression by promoting the genesis and recruitment of MDSC or by inducing the differentiation of TAMs into a tolerogenic phenotype [274]. CR and FMD suppress inflammation and reduce oxidative damage in humans and in preclinical mouse models [232,275]. Furthermore, we show that FMD reduces the levels of inflammatory markers such as the NLRP3 inflammasome, leukotriene in the heart of mice bearing melanoma and lung cancer and treated with immune checkpoint inhibitors [205,276].

Fasting improves chemotherapy efficacy against fibrosarcomas, mammary carcinomas, and non-small cell lung cancers by boosting the T-cell-mediated immune response, which leads to Treg depletion through enhanced ATP release due to fasting-induced autophagy in the tumor [273].

The effects of calorie restriction and diet on immune system activation could also be mediated through the reduction of high mobility group box protein 1 (HMGB1) [277].

HMGB1 is a multifunctional redox sensitive protein secreted by innate immune system cells (macrophages and NK) in TME under stress or inflammatory conditions. Extracellular HMG1 can play both protumor and antitumor roles. Reduced HMGB1 promotes immune cell activation and chemotaxis by stimulating the synthesis and release of proinflammatory cytokines, such as TNFα and IL1,6, through activation of the receptor for advanced glycation end products (RAGE) and/or Toll-like receptor (TLR) signals 2, 4 [278]. On the other hand, oxidized HMGB1, mostly present in the interstitial fluid of TME, inhibits DC activation and renders them tolerogenic via RAGE activation [279].

In addition to modulating the immune response, HMGB1 inhibits tumor growth and promotes the death of some tumor forms (as colorectal cancer) by inducing a metabolic shift towards anaerobic glycolysis through the inhibition of PKM2, an enzyme involved in the conversion of phosphoenolpyruvate into pyruvate, essential for the fuel of TCA and oxidative phosphorylation [280].

It has recently been shown that inhibition of extracellular HMGB1 blocks the growth of breast tumors through activation of the adaptive immune system and improves efficacy of immune checkpoint therapy (PD-1) by reducing the percentage of Tregs and increasing the percentage of M1 macrophages and activated DCs [281].

Recently it is emerging that the effects of fasting mimicking diet and caloric restriction on the activation of the antitumor immune response could be mediated by the intestinal microbiota remodeling [282]. Several studies have shown that the efficacy of chemotherapy and immunotherapy depend on the abundance and presence of bacterial species in the intestinal microbiota capable of secreting short-chain fatty acids (SCFA: acetic acid, propionic acid, butyric acid) [283,284]. The release of butyric acid inhibits histone deacetylases (HDACs), prevents CD8 exhaustion, and promotes effector CD8 activation by increasing IL12Rb expression through upregulation of the transcriptional regulator ID2 [285]. Calorie restriction exerts the antitumor response against colon and breast cancer by promoting CD8 activation through the enrichment of the microbiota with SCFA producing microbial families (butyric acid and acetic acid) [282].

At the same time, the composition of the microbiota could also influence the activation of the immune system directly through the production of serotonin [286] and indirectly through the release of SCFA, which promotes the transcription of tryptophan hydroxylase 1 (TPH1), the rate-limiting enzyme of the serotonin biosynthetic pathway, and colon serotonin production by enterochromaffin cells [287]. Serotonin is synthesized from tryptophan and, in addition to its crucial role as a mediator between the gut and the brain, plays an important role in regulating the immune system and tumor progression.

In fact, serotonin mitogenic property favors the progression of cancer [288]. On the other hand, serotonin signaling stimulates T cell activation and proliferation, promotes DC maturation, supports B cell development, enhances NK cell cytotoxicity, and stimulates macrophage polarization towards the M2 phenotype, while inhibiting M1 macrophage polarization [289,290]. However, the conversion of tryptophan to serotonin or kynurenine leads to tryptophan depletion in TME and thus to T-cell exhaustion and immune evasion. Although serotonin plays contradictory roles in regulating the functions of different immune cells, serotonin may have a pro-tumorigenic effect and enhance tumor immune evasion by generating an anti-inflammatory microenvironment.

Furthermore fasting/FMD cycles reverse chemotherapy-induced immunosuppression by promoting hematopoietic stem cell self-renewal (HSC) [291] and enhances cancer immune surveillance by improving memory T cell survival [292]. Indeed, fasting/calorie restriction boosts secondary immune response as it promotes memory T cell migration into the bone marrow where they meet a favorable microenvironment favorable to their survival and protection against chemotherapy [10,291]. Furthermore, T cells subjected to fasting acquire stem features as the lack of nutrients leads to an increase in autophagy and mitochondrial metabolism which causes a reduction in metabolic cofactors, such as acetyl coenzyme A, essential for epigenetic remodeling and cell differentiation. At the same time, the reduction of methionine and its intermediates affects histone methylation and compromises the activation of signaling pathways involved in stemness suppression [293].

Fasting/FMD cycles promote the selective expansion of early/progenitor exhausted effector T cells at the expense of late exhausted effector T cells in breast cancer [257]. Early exhausted effector T cells represent a subpopulation of exhausted/dysfunctional T cells, which correlate with the success of immune checkpoint inhibition and increased patient survival [294,295]. This subpopulation expresses dysfunctional markers such as TOX and PD1, but also exhibit self-renewing capacity and features of memory T cells, associated with the expression of transcriptional regulator T cell factor 1 (TCF1) [296,297,298]. Immune checkpoint blockade reverses immunosuppression and boosts immune response by increasing early exhausted effector T cell proliferation and differentiation in effector T cells, essential for long-term maintenance of persistent T cell responses. mTOR inhibition expands early exhausted effector T cells ex vivo and enhances long term T cell response and the efficacy of immune checkpoint inhibition [299]. Therefore, the frequency of early exhausted T cells is considered to be a predictive biomarker for favorable clinical outcome of checkpoint therapy.

Early exhausted effector T cells display higher mitochondrial mass and better mitochondrial fitness compared with late exhausted effector T cells. These mitochondrial changes are associated with high OXPHOS capacity, essential for supporting the early exhausted effector T cells’ self-renewal [299]. Fasting/FMD promotes the selective expansion of early exhausted effector T cells at the expense of late exhausted effector T cells in the breast cancer model by shifting the metabolism from glycolysis to OXPHOS and increasing b-oxidation of fatty acids [257].

Fasting favors the accumulation and activation of gd T cells in breast tissue and strengthens the anti-tumor immune response of cytotoxic CD8 T cells by producing ketone bodies and inhibiting mTOR activity. γδ T cells, particularly the Vd1^+^ subtype, can orchestrate an effective anti-tumor response, as supported by the positive correlation of their presence with better prognosis in TNBC patients [300]. Notably, ketone bodies produced by a ketogenic diet have been shown to promote the expansion and activation of gd T cells in the visceral mass [301], while the mTOR inhibition impairs the development of ab T cells but promotes gd T cell generation in the thymus [243,302].

Short-term fasting attenuates monocyte metabolic and inflammatory activity and inhibits monocyte mobilization from the bone marrow through suppression of systemic CCL2 production, leading to a drastic reduction of circulating monocytes that could affect infiltration and the composition of the tumor microenvironment [303]. Furthermore, fasting/FMD cycles reduce the accumulation of immunosuppressive polymorphonuclear (PMN) MDSC in TME, thereby enhancing the response to immunotherapy in breast cancer [257]. Fasting could enhance the efficacy of immune-checkpoint blockade through the production of ketone body, 3-hydroxybutyrate, as demonstrated for ketogenic diet, by preventing the upregulation of PD-L1 on myeloid cells and by expanding CXCR3^+^ T cells [270].

Fasting/FMD cycles also could enhance the immune response of anticancer therapies by promoting tumor vessel normalization and a reduction in size, number, and density by repressing the secretion of pro-angiogenic factors such as VEGF, factor VIII, inter- leukin-6 [IL-6], TNF-α, and plasminogen activator inhibitor-1 [PAI- 1] in breast cancer [257,265,266,304]. In addition, CR and fasting reduces fibroblast collagen deposition in the TME and cancer fibrosis by inhibiting, respectively, mTOR and TGF-β signaling, which promotes the conversion of fibroblast in cancer associated fibroblast (CAF) [305,306,307,308].

The less dense and viscous stroma, generated by CR and fasting, reduces the secretion of pro-inflammatory and pro-fibrotic cytokines, allows the infiltration of immune cells into the TME, and consequently enhances the diffusion of the anticancer therapy, thus enhancing the efficacy of anticancer treatment in breast cancer model [257,309].

## 8. Conclusions

Tumor cells alter the tumor microenvironment and help to create an immunosuppressive TME by restricting nutrients availability and by releasing metabolites, chemokines, and growth factors that reprogram the normal cell function and metabolism in TME, promote the recruitment of immunosuppressive cells and their differentiation in immune cell subtypes with immunosuppressive activity, and inhibit the activation of cytotoxic T lymphocytes and NK cells.

To meet metabolic demands and sustain a continuous, high proliferation rate, cancer cells rely mainly on glycolysis and glutamine catabolism as they generate intermediate metabolites that supply the amino acids, lipids, and nucleotides synthesis pathway and contribute to the synthesis of molecules involved in redox homeostasis maintenance. High tumor metabolic rate leads to glucose, glutamine, and amino acids (methionine, arginine, tryptophane, leucine, serine) depletion and the secretion of immunosuppressant lactate in TME. To support proliferation and effector response, activated T and NK cells need environmental nutrients, including glucose and amino acids, essential to promote PI3K/AKT/mTOR anabolic pathway activation. Therefore, the scarcity of nutrients, high lactate concentration, and low pH in TME lead to cytotoxic T and NK cells exhaustion and promote the proliferation of immune cells with immunosuppressive activity as their metabolism is more suited to this environment.

At the same time, tumor cells release chemokines and growth factors that promote tumor angiogenesis, the recruitment of immunosuppressive cells, such as MDSCs and M2 macrophages, and collagen deposition via fibroblast, making the extracellular matrix dense and rigid and further impeding T lymphocyte infiltration. Therefore, TME becomes a complex milieu of immune and normal cells and acellular components which support angiogenesis, tumor progression, and immune evasion.

CR, fasting, and FMD may reverse the immunosuppressive TME by affecting the cancer and immune cells’ metabolism, regulating growth factors and inflammatory mediators, and reshaping the extracellular matrix. The reduction of glucose and glutamine induced by caloric restriction, fasting, and FMD mainly affects the survival and proliferation of tumor cells as they are unable to shift their metabolism from glycolysis to oxidative phosphorylation and adapt to the new restrictive conditions. Furthermore, CR-dependent downregulation of stearoyl-CoA desaturase (SCD) limits the synthesis of monounsaturated fatty acids, essential for cell membrane fluidity and synthesis.

The reduction of IGF-1 in response to caloric restriction inhibits the PI3K-AKT-mTOR pathway and thus reduces the proliferation of tumor cells, immunosuppressive Tregs, and collagen deposition in TME and promotes normalization of tumor vasculature.

The metabolic changes induced by caloric restriction in T lymphocytes reshape the epigenetic landscape by reducing the production of acetyl coenzyme A and the expression of immune checkpoint receptors such as PD1. These metabolic and epigenetic changes improve T cell persistence, multipotency, tumor clearance, and their responsiveness to immunotherapy. Overall, calorie restriction promotes several changes in tumor cells, the immune system, and the extracellular matrix that may sensitize tumor cells to immunotherapy and enhance the immune response. However, a more in-depth analysis and detailed characterization of the effects of caloric restriction on tumor cells and TME is needed in order to develop, in the future, new dietary interventions that may have a greater synergistic effect with targeted anticancer therapies, minimizing the adverse or counterproductive effects induced by caloric restriction.
